# Association between amyloid-β42 levels and neuropsychiatric symptoms in Alzheimer’s disease trials

**DOI:** 10.1093/braincomms/fcaf089

**Published:** 2025-02-23

**Authors:** Jesus Thomas Abanto, Alok K Dwivedi, Bruno P Imbimbo, Alberto J Espay

**Affiliations:** James J. and Joan A. Gardner Family Center for Parkinson’s Disease and Movement Disorders, Department of Neurology, University of Cincinnati, Cincinnati, OH 45219, USA; Division of Biostatistics and Epidemiology, Department of Molecular and Translational Medicine, Texas Tech University Health Sciences Center, El Paso, TX 79905, USA; Research and Development Department, Chiesi Farmaceutici, Parma 43122, Italy; James J. and Joan A. Gardner Family Center for Parkinson’s Disease and Movement Disorders, Department of Neurology, University of Cincinnati, Cincinnati, OH 45219, USA

**Keywords:** Alzheimer’s disease, monomeric amyloid-β, CSF Aβ42, neuropsychiatric symptoms, anti-amyloid-β monoclonal antibodies

## Abstract

Research on how Alzheimer’s disease drugs impact neuropsychiatric symptoms is limited. Given the link between changes in cerebrospinal fluid (CSF) amyloid-β42 (Aβ42) levels and cognitive and clinical outcomes after anti-Aβ treatments, we hypothesized a similar association exists with neuropsychiatric symptoms. We conducted a meta-analysis of anti-Aβ drugs clinical trials to evaluate whether the changes in cerebrospinal Aβ42 levels are associated with neuropsychiatric symptoms, as measured by the Neuropsychiatric Inventory and if any such effect is mediated by changes in cognitive performance, as measured by the Mini-Mental State Examination. Data from 10 trials involving 10 746 Alzheimer’s disease patients were included. Decreases in Aβ42 levels were associated with worsening Neuropsychiatric Inventory scores (regression coefficient: −0.68; 95% confidence interval: −1.07 to −0.29; *P* = 0.002), and this association persisted after adjusting for Mini-Mental State Examination. Sensitivity analyses confirmed the robustness of these findings. Changes in CSF Aβ42 levels are inversely and independently associated with the frequency and severity of neuropsychiatric symptoms in anti-Aβ trials, suggesting a potential role of Aβ42 in modulating neuropsychiatric symptoms in Alzheimer’s disease.

## Teaser

Abanto *et al*. reported that the lower the cerebrospinal amyloid-β42 (Aβ42) levels, the worse the neuropsychiatric symptoms in patients with Alzheimer's disease who participated in anti-amyloid clinical trials. This suggests that strategies designed to increase Aβ42 levels could be beneficial in these patients.

## Graphical Abstract

**Graphical Abstract:**
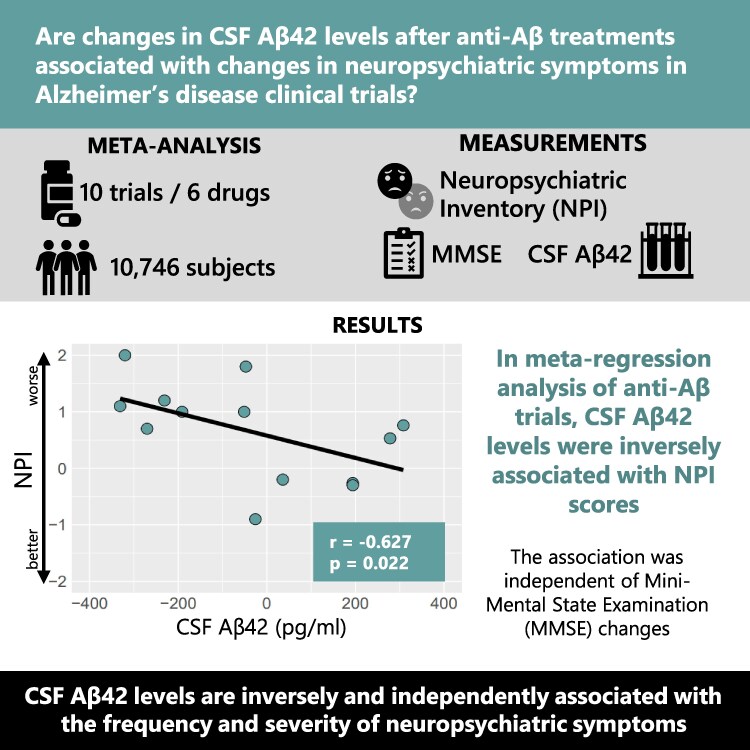

## Introduction

Neuropsychiatric symptoms (NPS) are considered precursors of incident mild cognitive impairment (MCI) and predict progression to Alzheimer's disease.^1^ NPS occur in the majority of patients with Alzheimer's disease. In a cross-sectional study involving 3608 participants, 43% of subjects with MCI and 75% with dementia exhibited NPS in the previous month, most commonly depression, apathy and agitation/aggression.^2^ Therapeutic approaches of NPS are crucial for the management of neurocognitive disorders. Anti-amyloid-β (Aβ) drugs are being developed to treat Alzheimer's disease and may affect the severity and frequency of NPS.

Limited information is available on the effects of anti-Aβ monoclonal antibodies (aducanumab, lecanemab and donanemab) on Alzheimer's disease-associated NPS. Some anti-Aβ drugs, especially β-secretase inhibitors, worsened NPS and triggered suicidal ideation.^3^ It is unclear whether these detrimental effects on behaviour are specific to the pharmacological class or linked to their effects on Aβ levels in the cerebrospinal fluid (CSF). There are conflicting studies evaluating the association between CSF Alzheimer's disease biomarkers and neuropsychiatry symptoms, as a review of 21 studies demonstrated.^4^ This review found that agitation/aggression was significantly and consistently related to core Alzheimer's disease CSF biomarkers, while depression was the only NPS occasionally associated with lower core Alzheimer's disease CSF pathology. A large study involving 1667 subjects on the Alzheimer's disease continuum reported that the severity of NPS, assessed using the Neuropsychiatric Inventory (NPI) Questionnaire Quick Version, was associated with Aβ42 levels but not with t-tau or p-tau.^5^ In a cross-sectional study of 445 MCI and Alzheimer's disease subjects, an inverse association was observed between CSF Aβ42 levels and NPI scores.^6^ Similarly, another cross-sectional study involving 784 cognitively normal and MCI subjects found that lower CSF Aβ42 levels were associated with more severe NPS, including anxiety, apathy and nighttime behaviour, as measured by the Neuropsychiatric Inventory Questionnaire Quick Version.^7^

We recently reported that post anti-Aβ treatment increases in CSF levels of Aβ42 improve cognitive and clinical endpoints whereas a decrease in this peptide worsens them.^8^ Extending the rationale of that study, we hypothesized that levels of CSF Aβ42 are associated with the severity and frequency of NPS following anti-Aβ drug treatments. The severity and frequency of NPS in clinical studies are typically measured using the NPI. We chose the Mini-Mental State Examination (MMSE) because it is the most common cognitive measure used in studies employing NPI to evaluate the cognitive status.^9,10^ Furthermore, unlike the Clinical Dementia Rating-Sum of Boxes or the Alzheimer's Disease Assessment Scale-Cognitive, the MMSE was the only instrument used in all anti-Aβ clinical trials. We sought to evaluate the associations between changes in CSF Aβ42 and NPS, as measured by the NPI, in long-term randomized trials of anti-Aβ drugs and determine whether any association would be mediated by cognitive changes, as measured by the MMSE.

## Materials and methods

### Search strategy

A comprehensive PubMed search was conducted to identify anti-Aβ drug trials in subjects with early, mild or mild-to-moderate Alzheimer's disease, published between January 1985 and August 2024, that reported data on CSF Aβ42, NPI (range = 0 to 120–144, depending on the 10- or 12-item NPI versions; higher scores indicate greater severity or more frequent occurrence of symptoms) and MMSE (range = 0 to 30; lower scores indicate worse cognitive status). Search terms included ‘Alzheimer’s Disease’ AND ‘clinical trial’ AND ‘cerebrospinal fluid’. PRISMA 2020 guidelines were followed.

### Eligibility criteria

Eligibility criteria included controlled trials of anti-Aβ drugs, with at least a 1-year follow-up, and reported CSF Aβ42, NPI and MMSE data. The primary outcomes were the difference in post- to pre-intervention changes in NPI and MMSE scores, and the primary exposure was changes in CSF Aβ42 levels between drug- and placebo-treated groups.

### Data extraction

Data were extracted from the article’s main text, tables and Supplementary material including patient population, interventions, doses, sample sizes, exposure times and CSF Aβ42 assays. If any data were only available in figures, values were extracted using WebPlotDigitizer. The resulting data set was double-checked by three authors (J.T.A., A.K.D. and B.P.I.).

### Quality assessment

Methodological quality was assessed using the National Heart, Lung, and Blood Institute (NHLBI) tool. Two authors (J.T.A. and A.K.D.) graded articles independently and disagreements were resolved by consensus.

### Statistical analysis

We used the placebo-adjusted mean change as the difference in the mean change in CSF Aβ42, NPI and MMSE from baseline between treatment and placebo groups. We assessed the association between placebo-adjusted changes in CSF Aβ42 and placebo-adjusted changes in NPI using restricted maximum likelihood random-effects meta-regression analyses. A weight was computed and assigned using the inverse variance of the placebo-adjusted mean differences in NPI. We used the *z*-standardized CSF Aβ42 in analysis by using the overall mean and standard deviation (SD) of the changes in CSF Aβ42 across studies. The estimated regression coefficient (RC) values by meta-regression analysis reflect the change in NPI or MMSE outcomes associated with a 1 SD increase in placebo-adjusted changes in CSF Aβ42. An *I*² statistic was used to evaluate heterogeneity in estimated associations between studies. The presence of publication bias and small sample size effects for NPI or MMSE were assessed using Funnel plots and Begg’s test, respectively. We also evaluated differences in the placebo-adjusted changes in NPI outcome between CSF Aβ42 groups (<0 versus ≥0) by applying a restricted maximum likelihood random-effects meta-regression. We calculated the weighted correlation coefficient (*r*) between changes in CSF Aβ42 and changes in NPI using fixed-effects meta-regression analysis. In fixed-effects models, the weight was computed using the sample sizes from each study for NPI assessment. Sensitivity analyses for random-effects models were performed after removing studies with increased heterogeneity in the associations, restricting analyses to large or unique studies. Sensitivity analysis was also performed for fixed-effects meta-analysis after accounting for robust variance estimation. The results of fixed or random-effects meta-regression analyses were summarized with RC, 95% CI and *P*-values. Statistical analyses were conducted using STATA 17.0, applying statistical checklists.

## Results

**Figure 1:**
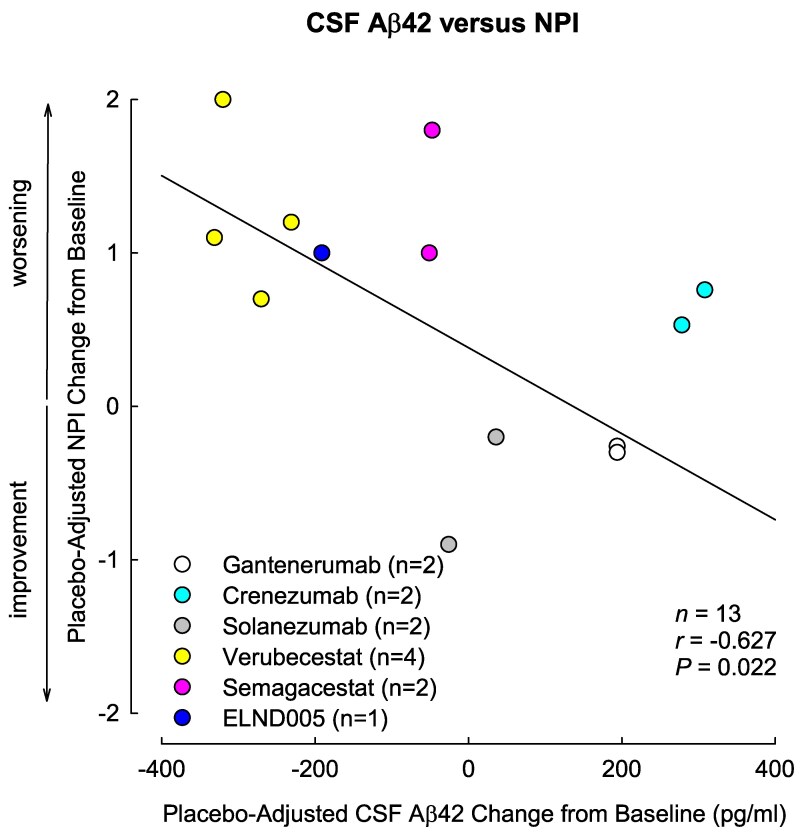
**Weight-adjusted correlation between placebo-adjusted CSF Aβ42 changes and placebo-adjusted changes in NPI across 13 trials of 6 anti-Aβ treatments (three monoclonal antibodies, one β-secretase inhibitor, one γ-secretase inhibitor and one Aβ aggregation inhibitor).** Individual studies are reported with different colours. A fixed-effects meta-regression analysis was applied to estimate the correlation coefficient (*r*) and test the associations between placebo-adjusted CSF Aβ42 changes and placebo-adjusted changes in NPI scores. Aβ42, 42-amino acid isoform of amyloid-β; CSF, cerebrospinal fluid; NPI, Neuropsychiatric Inventory.

We included 10 746 Alzheimer's disease subjects from 13 data sets derived from 10 unique trials (Table 1; Supplementary Fig. 1).^11-18^ Female participants accounted for 54.5% of all participants (54% in the placebo group and 55% in the drug group). There was no indication of publication bias (Supplementary Fig. 2) or small-study effect (*P* = 1.00 for NPI, *P* = 0.90 for MMSE). All included articles were of good or fair quality except for dropout rates > 20% during the follow-up period (Supplementary Table 1). In random-effects analysis, decreases in CSF Aβ42 were associated with worsening NPI scores (RC: −0.68; 95% CI: −1.07 to −0.29; *P* = 0.002, *I*² = 0%) without any heterogeneity. Moreover, placebo-adjusted changes in CSF Aβ42 (RC: −0.67; 95% CI: −1.10 to −0.24; *P* = 0.006) were found to be associated with changes in NPI, independent of placebo-adjusted MMSE changes. In the categorized analysis, negative CSF Aβ42 changes were associated with marked worsening in NPI scores (RC: −1.39; 95% CI: −2.13 to −0.66; *P* = 0.002, *I*² = 0%) without any heterogeneity (Table 2). In fixed-effects meta-regression analyses, reductions in CSF Aβ42 were associated with worsening NPI scores (*r* = −0.627, *P* = 0.022) (Fig. 1). Sensitivity analyses yielded similar results (Supplementary Table 2). Random-effects meta-analysis showed no association between placebo-adjusted changes in CSF Aβ42 and placebo-adjusted changes in MMSE scores (Supplementary Table 3). We did not observe any differences in the association between NPI and CSF Aβ42 by sex or drug class (Supplementary Table 4).

**Table 1:** Main characteristics of the studies included in this systematic analysis

Drug name	Dose	Subjects	Time (weeks)	Sample size		Placebo-adjusted change from baseline	
				Placebo	Drug	CSF Aβ42 (pg/ml)	NPI scores
Monoclonal antibodies							
Solanezumab^5^	400 mg/4 weeks	Mild/mod Alzheimer’s disease	80	506	506	−25.8	−0.9
Solanezumab^5^	400 mg/4 weeks	Mild/mod Alzheimer’s disease	80	519	521	36.1	−0.2
Crenezumab^6^	60 mg/kg/4 weeks	Early Alzheimer’s disease	105	409	409	278.2	0.5
Crenezumab^6^	60 mg/kg/4 weeks	Early Alzheimer’s disease	53	399	407	308.3	0.8
Gantenerumab^7^	510 mg/2 weeks	Mild Alzheimer’s disease	116	485	499	194	−0.3
Gantenerumab^7^	510 mg/2 weeks	Mild Alzheimer’s disease	116	477	498	194	−0.3
γ-Secretase inhibitors							
Semagacestat^8,9^	100 mg/day	Mild/mod Alzheimer’s disease	52	501	506	−51.1	1
Semagacestat^8,9^	140 mg/day	Mild/mod Alzheimer’s disease	52	501	527	−47.5	1.8
β-Secretase inhibitors							
Verubecestat^10^	12 mg/day	Mild/mod Alzheimer’s disease	78	653	652	−270.3	0.7
Verubecestat^10^	40 mg/day	Mild/mod Alzheimer’s disease	78	653	652	−331.3	1.1
Verubecestat^11^	12 mg/day	Prodromal Alzheimer’s disease	104	485	485	−231.1	1.2
Verubecestat^11^	40 mg/day	Prodromal Alzheimer’s disease	104	485	484	−320.2	2
Aβ aggregation inhibitors							
ELND005^12^	250 mg/bid	Mild/mod Alzheimer’s disease	78	82	84	−191.3	1

Mild/mod Alzheimer’s disease: Mild to moderate dementia due to Alzheimer’s disease/Prodromal Alzheimer’s disease: MCI due to Alzheimer’s disease/Early Alzheimer’s disease: MCI and mild dementia due to Alzheimer’s disease/Mild Alzheimer’s disease: Mild dementia due to Alzheimer's disease/CSF: cerebrospinal fluid; Aβ42: Aβ 42-amino acid isoform of amyloid-β/NPI: Neuropsychiatric Inventory.

**Table 2:** Random-effects analysis for evaluating the associations between placebo-adjusted changes in CSF Aβ42 and placebo-adjusted changes in NPI

	*N*	RC	95%CI		*P*-value	*I* ^2^
Continuous CSF Aβ42						
CSF Aβ42	13	−0.68	−1.07	−0.30	0.002	0%
Categorized CSF Aβ42						
CSF Aβ42 < 0 versus ≥0	13	−1.39	−2.13	−0.66	0.002	0%
Adjusted association						
CSF Aβ42	13	−0.67	−1.10	−0.24	0.006	0%
MMSE	13	−0.59	−1.56	0.39	0.211	

Aβ42, Aβ42-amino acid isoform of amyloid-β; CI, confidence interval; CSF, cerebrospinal fluid; MMSE, Mini-Mental State Examination; NPI, Neuropsychiatric Inventory; RC, regression coefficient.

## Discussion

Reductions in CSF Aβ42 levels following anti-Aβ treatments are associated with worse NPS and independently of cognitive changes. These clinical trial-based data are in agreement with three large observational cross-sectional studies in the Alzheimer's disease continuum, which showed that lower CSF Aβ42 levels were associated with higher NPI scores.^6,7,19^

The eligible trials included three monoclonal antibodies, one β-secretase inhibitor (verubecestat), one γ-secretase inhibitor (semagacestat) and one Aβ aggregation inhibitor (scyllo-inositol). The monoclonal antibodies (solanezumab, crenezumab and gantenerumab) included in this study primarily act on soluble, oligomeric and fibrillary forms of Aβ. These six drugs target the Aβ cascade through different mechanisms. Secretase inhibitors block the production of both Aβ42 and Aβ40 from the amyloid precursor protein, while monoclonal antibodies selectively increase Aβ42 levels by targeting amyloid plaques, which are predominantly composed of Aβ42. Monoclonal antibodies may also raise Aβ42 levels by preventing its aggregation. Secretase inhibitors are known to worsen cognitive performance in Alzheimer's disease patients, and these effects have been attributed to off-target interactions.^16^ If this hypothesis is correct, these off-targets would play a more significant role in regulating cognition than Aβ. Instead, our analysis supports the hypothesis that the detrimental effects of β-secretase and γ-secretase inhibitors on NPS are due to their reduction of Aβ42 production from amyloid precursor protein, with the association between Aβ42 and NPI remaining significant even after adjusting for the drugs’ impact on cognition (MMSE).

The heterogeneity of monoclonal antibodies notwithstanding our analysis suggests that antibody-mediated changes in Aβ42 play a role in the treatment responses. The mechanism is unclear but some studies suggest a potential effect of the treatments on brain connectivity,^20^ with recent research identifying an association between NPS and dementia subtypes based on patterns of brain connectivity.^21^ Therapeutic interventions with secretase or BACE1 inhibitors, which shut down the production of native Aβ, may be detrimental due to the blockade of Aβ-associated compensatory brain network changes.^22^ Collectively, these data support testing whether therapeutic strategies aimed at increasing CSF Aβ42 levels could also improve NPS.

Our study has several limitations. CSF Aβ42 levels were measured using different assays across trials. Although we calculated placebo-adjusted changes, the variability in assay methods may have introduced non-linearities in the data. Another key limitation is the absence of NPI data linked with CSF Aβ42 measurements in long-term studies involving the Food and Drug Administration-approved drugs aducanumab, lecanemab and donanemab. While aducanumab and lecanemab are known to markedly increase CSF Aβ42 levels,^8^ the missing NPI data may have affected our analyses.

In conclusion, our findings suggest that treatment-induced reductions in CSF Aβ42 levels may exacerbate NPS in Alzheimer's disease and MCI patients providing a rationale for testing the clinical impact on NPS of strategies aimed at increasing CSF Aβ42.

## Supplementary Material

fcaf089_Supplementary_Data

## Data Availability

The data that support the findings of this study are available from the corresponding author upon reasonable request.

## References

[1] Salazar R, Dwivedi AK, Royall DR. Cross-ethnic differences in the severity of neuropsychiatric symptoms in persons with mild cognitive impairment and Alzheimer's disease. J Neuropsychiatry Clin Neurosci. 2017;29(1):13–21. 27417070 10.1176/appi.neuropsych.15120423

[2] Lyketsos CG, Lopez O, Jones B, Fitzpatrick AL, Breitner J, DeKosky S. Prevalence of neuropsychiatric symptoms in dementia and mild cognitive impairment: Results from the cardiovascular health study. JAMA. 2002;288(12):1475–1483. 12243634 10.1001/jama.288.12.1475

[3] Panza F, Lozupone M, Logroscino G, Imbimbo BP. A critical appraisal of amyloid-beta-targeting therapies for Alzheimer disease. Nat Rev Neurol. 2019;15(2):73–88. 30610216 10.1038/s41582-018-0116-6

[4] Showraki A, Murari G, Ismail Z, et al Cerebrospinal fluid correlates of neuropsychiatric symptoms in patients with Alzheimer's disease/mild cognitive impairment: A systematic review. J Alzheimers Dis. 2019;71(2):477–501. 31424398 10.3233/JAD-190365

[5] Zhang M, Cho Y-E, Moon C. The relationship between neuropsychiatric symptoms, nighttime behaviors, and Alzheimer’s disease CSF biomarkers. Innov Aging. 2021;5(Supplement_1):652–652.

[6] Zeng Q, Wang Y, Wang S, et al Cerebrospinal fluid amyloid-β and cerebral microbleed are associated with distinct neuropsychiatric sub-syndromes in cognitively impaired patients. Alzheimers Res Ther. 2024;16(1):69. 38570794 10.1186/s13195-024-01434-7 PMC10988961

[7] Krell-Roesch J, Rakusa M, Syrjanen JA, et al Association between CSF biomarkers of Alzheimer's disease and neuropsychiatric symptoms: Mayo Clinic study of aging. Alzheimers Dement. 2023;19(10):4498–4506. 35142047 10.1002/alz.12557 PMC10433790

[8] Abanto J, Dwivedi AK, Imbimbo BP, Espay AJ. Increases in amyloid-β42 slow cognitive and clinical decline in Alzheimer's disease trials. Brain. 2024;147(10):3513–3521. 39259179 10.1093/brain/awae216

[9] Eikelboom WS, van den Berg E, Singleton EH, et al Neuropsychiatric and cognitive symptoms across the Alzheimer disease clinical spectrum: Cross-sectional and longitudinal associations. Neurology. 2021;97(13):e1276–e1287. 34413181 10.1212/WNL.0000000000012598 PMC8480405

[10] Lo TWB, Karameh WK, Barfett JJ, et al Association between neuropsychiatric symptom trajectory and conversion to Alzheimer disease. Alzheimer Dis Assoc Disord. 2020;34(2):141–147. 31633557 10.1097/WAD.0000000000000356

[11] Doody RS, Thomas RG, Farlow M, et al Phase 3 trials of solanezumab for mild-to-moderate Alzheimer's disease. N Engl J Med. 2014;370(4):311–321. 24450890 10.1056/NEJMoa1312889

[12] Ostrowitzki S, Bittner T, Sink KM, et al Evaluating the safety and efficacy of crenezumab vs placebo in adults with early Alzheimer disease: Two Phase 3 randomized placebo-controlled trials. JAMA Neurol. 2022;79(11):1113–1121. 36121669 10.1001/jamaneurol.2022.2909 PMC9486635

[13] Bateman RJ, Smith J, Donohue MC, et al Two Phase 3 trials of gantenerumab in early Alzheimer's disease. N Engl J Med. 2023;389(20):1862–1876. 37966285 10.1056/NEJMoa2304430 PMC10794000

[14] Doody RS, Raman R, Farlow M, et al A Phase 3 trial of semagacestat for treatment of Alzheimer's disease. N Engl J Med. 2013;369(4):341–350. 23883379 10.1056/NEJMoa1210951

[15] Doody RS, Raman R, Sperling RA, et al Peripheral and central effects of γ-secretase inhibition by semagacestat in Alzheimer's disease. Alzheimers Res Ther. 2015;7(1):36. 26064192 10.1186/s13195-015-0121-6 PMC4461930

[16] Egan MF, Kost J, Tariot PN, et al Randomized trial of verubecestat for mild-to-moderate Alzheimer's disease. N Engl J Med. 2018;378(18):1691–1703. 29719179 10.1056/NEJMoa1706441 PMC6776074

[17] Egan MF, Kost J, Voss T, et al Randomized trial of verubecestat for prodromal Alzheimer's disease. N Engl J Med. 2019;380(15):1408–1420. 30970186 10.1056/NEJMoa1812840 PMC6776078

[18] Salloway S, Sperling R, Keren R, et al A Phase 2 randomized trial of ELND005, scyllo-inositol, in mild to moderate Alzheimer disease. Neurology. 2011;77(13):1253–1262. 21917766 10.1212/WNL.0b013e3182309fa5 PMC3179648

[19] Banning LCP, Ramakers I, Köhler S, et al The association between biomarkers and neuropsychiatric symptoms across the Alzheimer's disease spectrum. Am J Geriatr Psychiatry. 2020;28(7):735–744. 32088096 10.1016/j.jagp.2020.01.012

[20] Imbimbo BP, Pomara N. Drug-induced reductions in brain amyloid-β levels may adversely affect cognition and behavior by a disruption of functional connectivity homeostasis. Neurodegener Dis Manag. 2019;9(4):189–191. 31337272 10.2217/nmt-2019-0013

[21] Zhao K, Xie H, Fonzo GA, Carlisle NB, Osorio RS, Zhang Y. Dementia subtypes defined through neuropsychiatric symptom-associated brain connectivity patterns. JAMA Netw Open. 2024;7(7):e2420479. 38976268 10.1001/jamanetworkopen.2024.20479 PMC11231801

[22] Panza F, Lozupone M, Bellomo A, Imbimbo BP. Do anti-amyloid-β drugs affect neuropsychiatric status in Alzheimer's disease patients? Ageing Res Rev. 2019;55:100948. 31454563 10.1016/j.arr.2019.100948

