# Supplementary material for: Association between amyloid-β42 levels and neuropsychiatric symptoms in Alzheimer’s disease trials
Source: Brain Commun. 2025 Feb 23;7(2):fcaf089. doi: 10.1093/braincomms/fcaf089 (PMC11879397; doi:10.1093/braincomms/fcaf089)
Supplement: fcaf089_Supplementary_Data [file fcaf089_supplementary_data.zip › Supplementary_Materials.pdf]

Supplementary Material

Title: Association Between Amyloid-β42 Levels and Neuropsychiatric Symptoms in Alzheimer's Disease Trials

Table of contents

**Supplementary Figure 1.** Flowchart of study selection	**3**
**Supplementary Figure 2.** Funnel plots for NPI (upper panel) and MMSE (lower panel).	**4**
**Supplementary Table 1.** Quality appraisal of each eligible study using the Study Quality Assessment Tool developed by the National Heart, Lung and Blood Institute (NHLBI).	**6**
**Supplementary Table 2.** Associations between placebo-adjusted changes in CSF Aβ42 and placebo-adjusted changes in NPI using sensitivity analyses.	**7**
**Supplementary Table 3.** Random-effects meta-analysis for evaluating the associations between placebo-adjusted changes in CSF Aβ42 and placebo-adjusted changes in MMSE.	**8**
**Supplementary Table 4.** Subgroup analyses of the associations between placebo-adjusted changes in CSF Aβ42 and placebo-adjusted changes in NPI by drug type and sex percentages	**8**

Supplementary Figure 1: Flowchart of study selection**.**

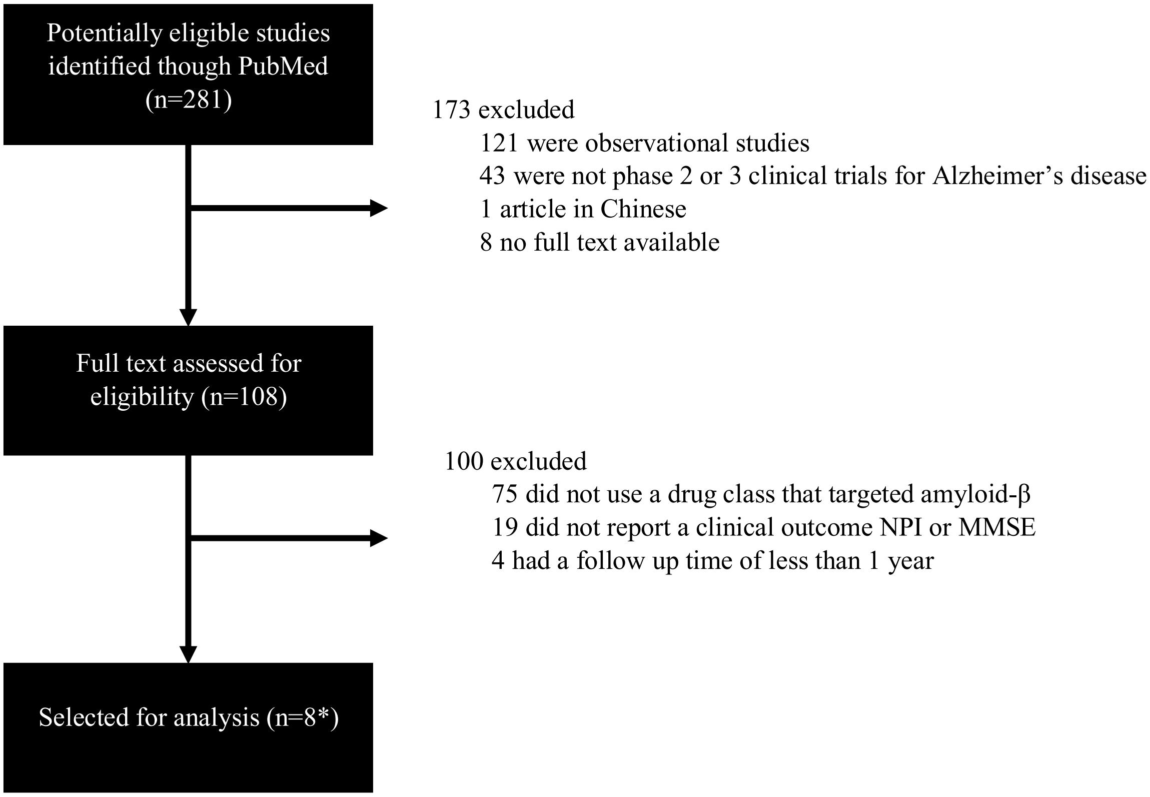

*These 8 studies included the results of 13 datasets from 10 trials.

Supplementary Figure 2. Funnel plots for NPI (panel A) and MMSE (panel B) for all 13 selected trials. Each point is a different trial. NPI: neuropsychiatric inventory; MMSE: Mini-Mental State Examination. *P*-values were calculated using Begg's test.

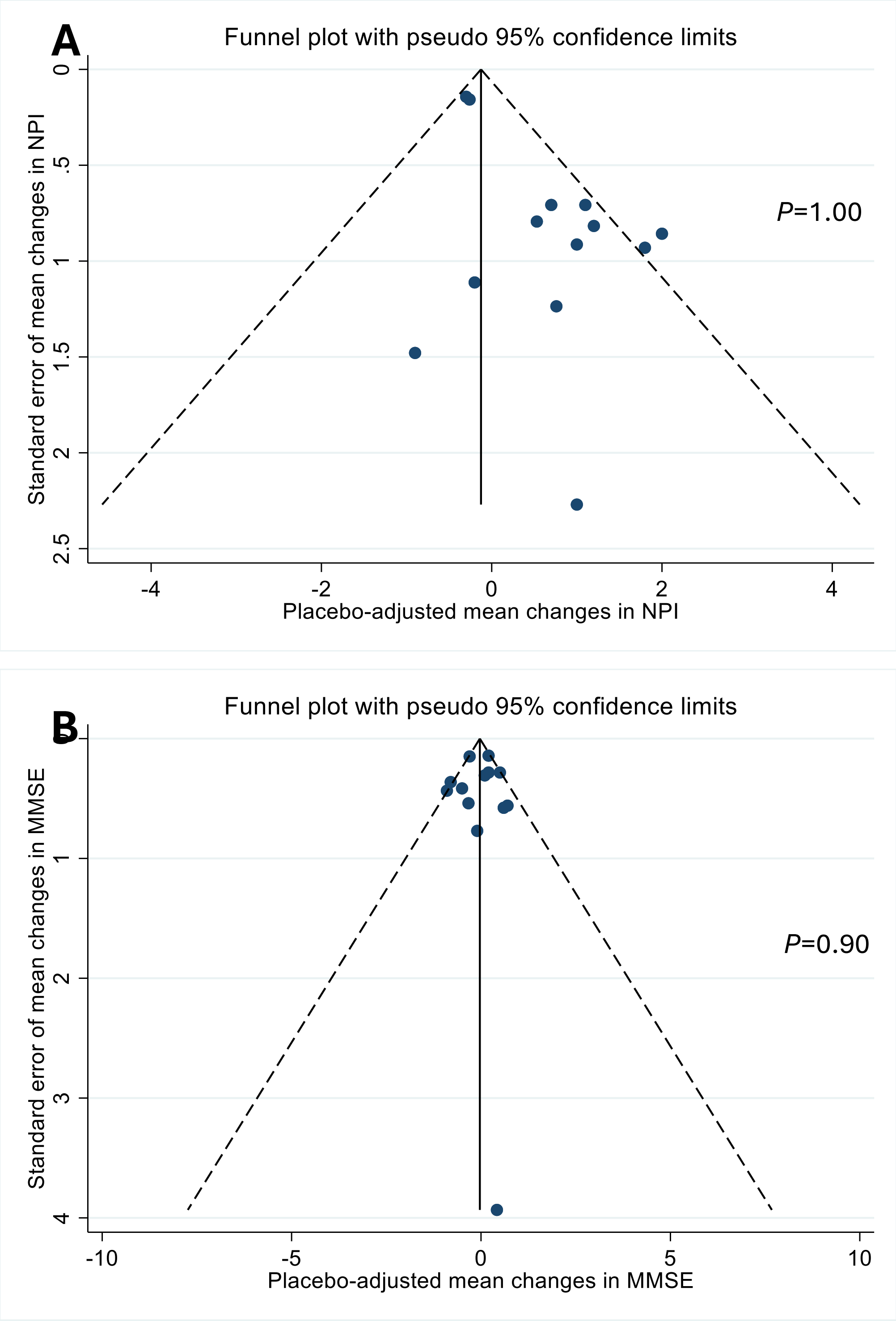

Supplementary Table 1. Quality appraisal of each eligible study using the Study Quality Assessment Tool developed by the National Heart, Lung and Blood Institute (NHLBI).

Reference	NCT	Item 1	Item 2	Item 3	Item 4	Item 5	Item 6	Item 7	Item 8	Item 9	Item 10	Item 11	Item 12	Item 13	Item 14
Doody et al, 2014[1]	**NCT00905372** **NCT00904683**	Yes	Yes	Yes	Yes	Yes	Yes	22 to 26%	0 to 4%	NA	Yes	Yes	Yes	Yes	Yes
Doody et al, 2013[2]; Doody et al, 2015[3]	**NCT00594568**	Yes	Yes	Yes	Yes	Yes	Yes	69 to 76%	5 to 14%	NA	Yes	Yes	Yes	Yes	Yes
Egan et al, 2018[4]	**NCT01739348**	Yes	Yes	Yes	Yes	Yes	Yes	29%	2%	NA	Yes	Yes	Yes	Yes	Yes
Egan et al, 2019[5]	**NCT01953601**	Yes	Yes	Yes	Yes	Yes	Yes	50 to 52%	2%	NA	Yes	Yes	Yes	Yes	Yes
Ostrowitzki et al, 2022[6]	**NCT02670083** **NCT03114657**	Yes	Yes	Yes	Yes	Yes	Yes	80 to 100%	20%	NA	Yes	Yes	Yes	Yes	Yes
Bateman et al, 2023[7]	**NCT03444870** **NCT03443973**	Yes	Yes	Yes	Yes	Yes	Yes	21.5%	8.5%	NA	Yes	Yes	Yes	Yes	Yes
Salloway et al, 2011[8]	**NCT00568776**	Yes	Yes	Yes	Yes	Yes	Yes	39.3%	4.5%	NA	Yes	Yes	Yes	Yes	Yes

Item 1: Was the study described as randomized, a randomized trial, a randomized clinical trial, or an RCT? / Item 2: Was the method of randomization adequate (i.e., use of randomly generated assignment)? / Item 3: Was the treatment allocation concealed (so that assignments could not be predicted)? / Item 4: Were study participants and providers blinded to treatment group assignment? / Item 5: Were the people assessing the outcomes blinded to the participants' group assignments? / Item 6: Were the groups similar at baseline on important characteristics that could affect outcomes (e.g., demographics, risk factors, co-morbid conditions)? / Item 7: Was the overall drop-out rate from the study at endpoint 20% or lower of the number allocated to treatment? / Item 8: Was the differential drop-out rate (between treatment groups) at endpoint 15 percentage points or lower? / Item 9: Was there high adherence to the intervention protocols for each treatment group? / Item 10: Were other interventions avoided or similar in the groups (e.g., similar background treatments)? / Item 11: Were outcomes assessed using valid and reliable measures, implemented consistently across all study participants? / Item 12: Did the authors report that the sample size was sufficiently large to be able to detect a difference in the main outcome between groups with at least 80% power? / Item 13: Were outcomes reported or subgroups analyzed prespecified (i.e., identified before analyses were conducted)? / Item 14: Were all randomized participants analyzed in the group to which they were originally assigned, i.e., did they use an intention-to-treat analysis? / NA: Not available

References:

1. Doody RS, Thomas RG, Farlow M, et al. Phase 3 trials of solanezumab for mild-to-moderate Alzheimer's disease. *N Engl J Med*. 2014;370(4):311-321. doi:10.1056/NEJMoa1312889
2. Doody RS, Raman R, Farlow M, et al. A phase 3 trial of semagacestat for treatment of Alzheimer's disease. N Engl J Med. 2013;369(4):341-350. doi:10.1056/NEJMoa1210951
3. Doody RS, Raman R, Sperling RA, et al. Peripheral and central effects of γ-secretase inhibition by semagacestat in Alzheimer's disease. Alzheimers Res Ther. 2015;7(1):36. doi:10.1186/s13195-015-0121-6
4. Egan MF, Kost J, Tariot PN, et al. Randomized Trial of Verubecestat for Mild-to-Moderate Alzheimer's Disease. N Engl J Med. 2018;378(18):1691-1703. doi:10.1056/NEJMoa1706441
5. Egan MF, Kost J, Voss T, et al. Randomized Trial of Verubecestat for Prodromal Alzheimer's Disease. N Engl J Med. 2019;380(15):1408-1420. doi:10.1056/NEJMoa1812840
6. Ostrowitzki S, Bittner T, Sink KM, et al. Evaluating the Safety and Efficacy of Crenezumab vs Placebo in Adults With Early Alzheimer Disease: Two Phase 3 Randomized Placebo-Controlled Trials. JAMA Neurol. 2022;79(11):1113-1121. doi:10.1001/jamaneurol.2022.2909
7. Bateman RJ, Smith J, Donohue MC, et al. Two Phase 3 Trials of Gantenerumab in Early Alzheimer's Disease. N Engl J Med. 2023;389(20):1862-1876. doi:10.1056/NEJMoa2304430
8. Salloway S, Sperling R, Keren R, et al. A phase 2 randomized trial of ELND005, scyllo-inositol, in mild to moderate Alzheimer disease. Neurology. 2011;77(13):1253-1262. doi:10.1212/WNL.0b013e3182309fa5

Supplementary Table 2. Associations between placebo-adjusted changes in CSF Aβ42 and placebo-adjusted changes in NPI using sensitivity analyses.

	N	RC	95%CI		*P* value
Fixed effects meta-analysis	13	-0.63	-1.16	-0.11	0.022
Fixed effects meta-analysis with robust variance estimation	13	-0.63	-0.97	-0.30	0.002
Random effects meta-analysis on unique studies	10	-0.75	-1.29	-0.21	0.012
Random effects meta-analysis on large studies (n >500)	10	-0.63	-0.99	-0.28	0.003

RC: regression coefficient; CI: confidence interval; NPI: neuropsychiatric inventory; CSF: cerebrospinal fluid; Aβ42: 42-amino acid isoform of amyloid-β

Supplementary Table 3. Random-effects meta-analysis for evaluating the associations between placebo-adjusted changes in CSF Aβ42 and placebo-adjusted changes in MMSE.

	RC	95%CI		*P* value	I^2
All studies	-0.04	-0.38	0.30	0.809	52.2%
After removing studies with high heterogeneity	-0.26	-0.56	0.05	0.087	15.4%

RC: regression coefficient; CI: confidence interval; MMSE: Mini-Mental State Examination; CSF: cerebrospinal fluid; Aβ42: 42-amino acid isoform of amyloid-β

Supplementary Table 4. Subgroup analyses of the associations between placebo-adjusted changes in CSF Aβ42 and placebo-adjusted changes in NPI by drug type and sex percentages

Subgroups	RC	95%CI		*P* value
Female>55%	-0.462	-1.205	0.282	0.171
Female <=55%	-0.442	-2.219	1.336	0.528
Monoclonal antibodies	0.360	-1.570	2.289	0.652
Non-monoclonal antibodies	0.092	-1.787	1.971	0.899

RC: regression coefficient; CI: confidence interval; NPI: neuropsychiatric inventory; CSF: cerebrospinal fluid; Aβ42: 42-amino acid isoform of amyloid-β
